# National Campaign to Prevent Falls in the Elderly Population September 27

**DOI:** 10.1016/S1808-8694(15)31083-1

**Published:** 2015-10-19

**Authors:** Fernando F. Ganança, Raquel Mezzalira, Oswaldo Laércio M. Cruz

**Affiliations:** 1 Adjunct Professor of Otology/Neurotology — Department of Otorhinolaryngology and Head and Neck Surgery — Federal University of São Paulo — Paulista School of Medicine. Campaign Coordinator; 2 M. Sc in Medical Sciences — University of Campinas Medical School — UNICAMP and attending physician — Department of Neurotology — Otorhinolaryngology and Head and Neck Surgery Course - UNICAMP. Campaign Coordinator; 3 Associate Professor — Otorhinolaryngology — University of São Paulo. Affiliate Professor, Head of of Otology/Neurotology Course — Otorhinolaryngology — Federal University of São Paulo. President of the Brazilian Society of Otology (SBO)

On September 27 we celebrate Elderly's Day, and the Brazilian Association of Otology (SBO) will once again hold the National Campaign to Prevent Falls in the Elderly Population.

There are many reasons behind this SBO's effort. Falls are the 6th main cause of death in people above 65 years of age^1^. It is estimated that 30% of the persons above this age range suffer falls at least once a year^2^, and these falls are responsible for 70% of the accidental deaths in individuals with 75 years or more^3^.

These estimates point to alarming figures, because in 2025, our country will have 31.8 million persons with 60 years or more, and will be the 6th country in the world in terms of elderly persons^4^.

Vestibular disorders have a particular importance in this context, because an increase in age is directly associated with the presence of different vestibular symptoms and clinical manifestations such as: vertigo and other types of dizziness, body balance disorders, movement restriction, physical and psychological insecurity to walk or gait disorders and falls^5^^,^^6^. These alterations in the labyrinth increase the number of falls in elderly patients in relation to their healthy counterparts^7^.

Based on this data, the SBO launched on September 27^th^ of 2005, the National Campaign to Prevent Falls in the Elderly, aiming at educating the elderly population about the risk of falls and how to prevent them. So far we had been doing an educational work so that people could identify and eliminate major risk factors. Such measure is fundamental because about 60% of falls happen at home, during daily activities, and 25% of them result from “domestic hazards”, such as slippery floors, inadequate lighting, mal-positioning of furniture. The bedroom-to-bathroom route, especially at night, is considered the highest risk of household-related falls. While some risk factors for falls can not be changed, with aging, others can be easily eliminated or reduced.

Now, with the “A day to care for the elderly with dizziness”, the SBO will start the second stage of the campaign. This event will be held on September 27 (Elderly's Day) and is organized in such a way as to collect epidemiological data to assess the occurrence of vestibular disorders, past falls, risk of future falls, among other socio-demographic and clinical data. The clinical and socio-demographic information are collected through the patient-interview protocol created by the SBO, which will be made available soon at the website www.sbotologia.com.br/campanhaquedas.

Besides initial care to the patient and data collection, the effort here aims at continuing to educate the general public about the problems associated with falls in the elderly, stimulate those elderly with dizziness and/or past history of falls to look for otorhinolaryngological care, referring them to diagnostic procedures and treatment of the possible vestibular disorders, and also to educate them about prevention. We will hand out information about dizziness and falls, by using the material created by the SBO.

This broad effort, spanning the entire country, will only be possible with the participation of all Otorhinolaryngology Clinics Certified by the Brazilian Association of Otorhinolaryngology and Neck and Facial Surgery (ABORL-CCF) and the physicians members of this association. The invitation will be made through e-mail, to the members of the association who are duly enrolled.

Those already interested in participating may sign up in the website http://www.sbotologia.com.br/campanhaquedas/seu-cadastro.asp and define the number of patients for this first care until May 30^th^, 2008. This service will be rendered free of charge to the patient. We must also stress that this first care will find some patients with vestibular disorders. Thus, the colleagues and clinics who choose to participate in this first care must commit themselves to the later clinical follow up of the patients (diagnostic investigation and treatment) of the elderly with vestibular disorders.

The campaign will be announced by the SBO in the specialized media (radio, newspaper and television), however the members of our association or certified clinic may also help with this disclosure in their communities.

After the event, the SBO intends to collect all the information obtained for analysis and later disclosure, following a multicentric epidemiologic clinical study.

For further information, access our website.

For this campaign to be successful, the SBO counts on your participation.
